# Loading rate, geometry, and damage state influence vertical extraction biomechanics in an ex vivo swine dental model

**DOI:** 10.3389/fbioe.2024.1491834

**Published:** 2025-01-07

**Authors:** Timothy J. Gadzella, Karyne N. Rabey, Michael R. Doschak, Lindsey Westover, Owen Addison, Dan L. Romanyk

**Affiliations:** ^1^ Department of Mechanical Engineering, University of Alberta, Edmonton, AB, Canada; ^2^ Department of Surgery, University of Alberta, Edmonton, AB, Canada; ^3^ Faculty of Pharmacy and Pharmaceutical Sciences, University of Alberta, Edmonton, AB, Canada; ^4^ Faculty of Dentistry, Oral and Craniofacial Sciences, King’s College London, London, United Kingdom; ^5^ School of Dentistry, University of Alberta, Edmonton, AB, Canada

**Keywords:** tooth extraction, periodtonal ligament, viscoelacity, tissue damage, tissue mechanics

## Abstract

**Introduction:**

Validated models describing the biomechanics of tooth extraction are scarce. This study seeks to perform experimental and numerical characterization of vertical tooth extraction biomechanics in swine incisors with imposed vertical extraction loads. Imaging analysis related mechanical outcomes to tooth geometry and applied loading rate. Then, the predictive capabilities of the developed finite element analysis (FEA) models were demonstrated by testing different loading scenarios and validating the results against experimental equivalents.

**Methods:**

Simulated vertical extractions were performed on partial swine central incisors (n = 49) and studied for peak extraction force and dental complex stiffness. Post-extraction µCT images were obtained to measure root surface attachment area (RSAA) and observe patterns of periodontal ligament (PDL) rupture. Crosshead force-displacement data was used in an inverse finite element analysis (IFEA) to verify parameters for the PDL in an axisymmetric model of tooth extraction. New force-hold loading protocols were devised *in silico* and validated in a series of tests on swine incisors to demonstrate the predictive efficacy of the finite element model. Force-hold loading on an initially-damaged PDL was also simulated.

**Results:**

Reductions in loading rate and RSAA were found to significantly reduce peak extraction forces by 98N–120 N. Increases in instantaneous stiffness during loading were associated with increases in loading rate. Inverse finite element solutions demonstrated consistent PDL parameters across loading cases. Force-hold loading predicted extraction behaviour with large variance in extraction time. Damage imposed in the FEA model was able to predict experimental results from experiments on similarly-damaged dental complexes.

**Conclusion:**

This study presents a comprehensive experimental and numerical characterization of vertical tooth extraction biomechanics employing an *ex vivo* swine model. The results of these experiments suggest that the axisymmetric FEA model is a powerful tool for predicting a range of conditions and dental complex geometries. The predictive power of the FEA model demonstrated in this study encourages its use in pre-clinical testing and development of new vertical extraction loading schemes for improving clinical outcomes.

## 1 Introduction

Tooth extraction is among the most common procedures in dental medicine with a range of causes and indications (e.g., tooth decay) (Broers et al., 2022a; Broers et al., 2022b; Hiltunen and Vehkalahti, 2023; Bernal-Sánchez et al., 2023; Alibrahim et al., 2023). Despite its importance to clinical practice, there is sparse literature available characterising the biomechanics of tooth extraction in clinical (Beuling et al., 2023; Ahel et al., 2006; Ahel et al., 2015; Lehtinen and Ojala, 1980; Dietrich et al., 2020), numerical (Genna and Paganelli, 2014; Gadzella et al., 2024), or experimental laboratory models (Genna and Paganelli, 2014; Gadzella et al., 2023; Chiba et al., 1980). The scarcity of biomechanical data contrasts with growing interest in atraumatic tooth extraction, a family of techniques that alter application of load to the extracted tooth to reduce the damage caused to the remaining bone and gingival tissue. Techniques for atraumatic extraction include the sectioning of teeth with piezoelectric or air-driven tools (Mohammadi, 2023; Muska et al., 2013; Hamed and Mohamed, 2023); severing the periodontal ligament (PDL) with elevators, luxators, or periotomes (Mohammadi, 2023; Muska et al., 2013; Hamed and Mohamed, 2023; Schropp et al., 2003; El-Kenawy and Ahmed, 2015; Papadimitriou et al., 2012; Sharma et al., 2015; Contractor et al., 2023); or application of novel loading devices such as Physics^®^ forceps (El-Kenawy and Ahmed, 2015; Patel et al., 2016; Irshad et al., 2023) or the Benex^®^ vertical tooth extraction device (Dietrich et al., 2020; Makki et al., 2021; Muska et al., 2013). Biomechanical data describing the forces or displacements applied in these studies is rarely collected despite the intentional changes each make relative to the biomechanics of conventional extraction, either in the mechanism by which loading is applied to the dental complex or the damage induced in the dental complex to assist in retrieval of the tooth root. The metrics by which these techniques are commonly assessed are clinical in nature and include measures such as socket depth or gingival rupture size (Mohammadi, 2023; Schropp et al., 2003; Sharma et al., 2015; Contractor et al., 2023; Makki et al., 2021; Hariharan et al., 2014; Patel et al., 2016; Irshad et al., 2023), self-reported pain (Contractor et al., 2023; Makki et al., 2021; Hariharan et al., 2014; Patel et al., 2016; Irshad et al., 2023), or procedure time (Muska et al., 2013; Contractor et al., 2023; Hariharan et al., 2014; Patel et al., 2016; Irshad et al., 2023). It is less common for alveolar bone resorption (Hamed and Mohamed, 2023; Schropp et al., 2003) or cellular response (Mohammadi, 2023; Tohar et al., 2022; Makki et al., 2021) to be measured following tooth extraction, although these metrics could most closely be associated with differences in biomechanical load based on the hypothesis that the loading nature determines the extent of the damage and subsequent resorption response.

The lack of congruency among biomechanical measurement and clinical outcomes is associated with the technical difficulty of collecting biomechanical data in clinical models of tooth extraction. Traditional forceps are difficult to instrument and limit the amount of data that can be gathered by measuring the forces applied to the dental complex during traditional tooth extraction. Methods such as attaching airbags (Ahel et al., 2015; Ahel et al., 2015) or strain gauges (Lethinen and Ojala, 1980) to forceps can distinguish between grip pressure and rotational moment, but do not provide directional data needed to understand how the force interacts with the dental complex. Vertical tooth extraction with the Benex device affords an opportunity to overcome such limitations because the only force intentionally applied to the tooth root is along its long axis and the configuration of the device’s components facilitates the inclusion of a load cell that can measure the extraction force (Dietrich et al., 2020). However, current extraction procedures are guided through a combination of haptic feedback mechanisms and clinician discretion (Dietrich et al., 2020; Muska et al., 2013) because of lacking biomechanical data related to clinical outcomes that may inform procedures in real time. Although the effectiveness of these atraumatic techniques has been studied on the basis of clinical outcomes, a link to foundational descriptive biomechanical data prevents further improvements to protocols and devices.

Laboratory-based experimental models for characterising biomechanics in the dental complex tend to focus on the mechanical behaviour of the PDL in small, isolated tissue sections in uniaxial loading regimes to inform constitutive models (Toms et al., 2002; Oskui and Hashemi, 2016; Huang et al., 2016; Jang et al., 2018; Lin et al., 2017; Najafidoust et al., 2023; Genna et al., 2008). The entire dental complex response has also been examined in occlusal-apical or buccal-lingual loading directions to replicate physiological or orthodontic loading (Lin et al., 2014; Papadopoulou et al., 2014; Bosiakov and Mikhasev, 2015; Romanyk et al., 2017; Knaup et al., 2018). Few experimental models focus on the load directions and displacement magnitudes required to characterize tooth extraction. Models of tooth extraction on the complete dental complex (Genna and Paganelli, 2014; Gadzella et al., 2023; Chiba et al., 1980; Knaup et al., 2018; Ansbacher et al., 2023) predominantly consider vertical tooth extraction because the loading vectors are known but have been limited in the range of load regimes studied (Gadzella et al., 2024; Ansbacher et al., 2023), sample size (Gadzella et al., 2023), and applicability to the human clinical model (Chiba et al., 1980). Recent experimental work has sought to improve on laboratory-based experimental methods for mounting *ex vivo* swine samples in testing apparati by introducing self-aligning motion elements to reduce off-axis forces acting on the tooth root (Gadzella et al., 2023). In general, these experiments are limited in their capacity to characterize the tooth geometry and its influence on the complex response as well as the mechanisms of tissue rupture underlying the overall dental complex response. Post hoc analyses of residual PDL have been performed in clinical models to examine cell viability for re-implantation (Baschong et al., 2018) and bone resorption (Heinonen et al., 2018), but the rupture behaviours of the PDL have not been assessed alongside a rigorous characterization of their underlying extraction mechanics.

The scarcity of biomechanical data drives limitations in numerical models that characterize the dental complex for understanding tooth extraction. Models that are suitable for understanding tooth extraction include high-strain formulations that account for tissue viscoelasticity and damage in the PDL (Gadzella et al., 2024; Natali et al., 2008; Ortún-Terrazas et al., 2019). However, these models suffer from high computational costs due to their reliance on subject-specific mesh generation and the application of custom solver code and material subroutines, making them difficult to use as generalized tools for studying tooth extraction procedures in a broader population. Recently, an axisymmetric model including PDL viscoelasticity, hyperelasticity, and damage was developed (Gadzella et al., 2024), showing promise for representing the biomechanics of tooth extraction, but was limited by a sparse supporting data set.

The current study addresses the limitations of experimental and numerical characterisation of tooth extraction by exploring a range of advanced, clinically-applicable loading schemes *ex vivo* and utilizing the biomechanical outcomes to drive numerical modeling advancements. Imaging of the post-extraction PDL provides new insight into its role in tooth extraction biomechanics. The collected data are then applied to study two new factors in tooth extraction. First, the prediction of dental complex behaviour under a force-controlled loading to a threshold force which is then maintained (“force-hold loading”) is demonstrated as an application of the generalizable numerical model. Second, the model is used to predict the behaviour of the dental complex under force-hold loading with imposed PDL damage. The objectives of this study are (1) to provide robust characterization of dental complex biomechanics under vertical extraction loading; and (2) to apply these characterizations to the prediction of novel, clinically-relevant loading scenarios and tissue damage conditions.

## 2 Methods

### 2.1 Mechanical tooth extraction experiments

The general approach to mechanically test *ex vivo* samples under simulated vertical tooth extraction and design of the experimental device has been presented and validated in a previous study (Gadzella et al., 2023). Briefly, the mandibles of juvenile swine were acquired under a secondary-use exemption provided by the University of Alberta Research Ethics Office (REO Reference No. ETR65) and cut *ex vivo* to isolate the central incisors at an approximate root length of 15 mm. Samples were then potted in dental stone and attached to a custom self-aligning apparatus within a material test frame (Instron E3000, Instron, Norwood, United States). Both central incisors from each specimen were extracted under various load rates and schemes to observe the effects of different loading on extraction mechanics. The tested load schemes were continuous displacement control at 0.2 mm/min and 2 mm/min, continuous force control at 10 N/min and 100 N/min, and intermittent displacement control at 2 mm/min with displacement-held dwell periods, with n = 10 samples per group. Twenty-five (25) central incisors were extracted in this study (5 per loading group) and the data combined with the results of a previous study on the same model (Gadzella et al., 2023) for a total of 50 simulated extractions. Extractions were determined to be successful if the incisor was removed from the socket without visually recognizable fracture to the tooth or surrounding bone.

The video-assisted stiffness analysis from previous work was repeated using load-displacement data from all successful continuous-loading extractions (Gadzella et al., 2023). Obtained curves were used to calculate the instantaneous stiffness through the loading periods of each extraction. Instantaneous stiffness curves were input to a K-means algorithm in MATLAB (Mathworks, 2006) to identify clusters in the stiffness data based on stiffness magnitude and curve shape. In doing so, the K-means algorithm presented patterns in the instantaneous stiffness while being blinded to the underlying load regime. The probability of a stiffness curve from an extraction at a given load rate being sorted into each curve was calculated after the K-means algorithm to assess if the patterns driving the cluster sorting corresponded to the loading rate and control scheme. The robustness of this method for sorting stiffness curves and their relationship to the underlying load rate is further detailed in Supplementary Material S1.

#### 2.1.1 Post-extraction imaging of tooth surfaces and residual PDL

Teeth from all successful extractions were scanned with x-ray microcomputed tomography (µCT) at a nominal resolution of 9 micron following extraction. Post-extraction images of the incisors were gathered for the measurement of tooth root surface attachment area (RSAA) as a tooth geometry. Contrast-enhanced (CE) images were also collected for a subset of teeth to provide 3D representations of the residual PDL that were examined for patterns or features that may relate to the mechanics of PDL rupture behaviour. All X-ray based computed tomographyimages were obtained in Skyscan 1076 and 1176 CT scanners with a 1 mm aluminum filter at 90kV and 100 µA of source voltage and current, respectively. All scans were reconstructed in the vendor supplied Skyscan NRecon v1.6 software with default levels of ring artifact, beam hardening, and smoothing corrections. An additional 21 incisors were imaged with a contrast-enhanced µCT protocol (CE- µCT) whereby they were fixed in 10% neutral buffered formalin and then stained in a 5% by-weight solution of mercury chloride in water for 24 h, followed by 24 h destaining in deionized water prior to µCT imaging. The remaining 20 incisors were imaged under the same imager settings at the same resolution but were only fixed in formalin prior to imaging.

Reconstructed µCT image stacks were cropped in FIJI ImageJ to exclude the volume surrounding the sample tubes in which the teeth were imaged and thereby reduce memory load before being imported to Materialise Mimics (Version 25, Materialise, Leuven, Belgium) for segmentation and analysis. Images were segmented semi-automatically using the region grow algorithm to capture the outer geometry of the tooth’s hard tissue surface. Three-dimensional open and close morphological operations were performed with 26-voxel connectivity to smooth the masks. Slice editing with interpolation was performed by hand where needed to improve the coverage of the segmentation masks where the region-growth algorithm had caused the masks to overlap at tissue boundaries or include small, disconnected bodies due to noise. This procedure was also applied to capture the geometry of the soft tissue in the CE-µCT scans.

Three-dimensional bodies were generated from the segmentation in MIMICS and were exported to 3-Matic (Version 17, Materialise, Leuven, Belgium) for RSAA measurement and examination of PDL rupture patterns. The freehand triangle selection tool was used to select the entire surface of the tooth below the boundary of PDL attachment. The resulting surface was exported to a separate part, the surface area of which was taken to be the RSAA for that tooth. Soft tissue bodies from CE-µCT scans were examined to qualitatively identify patterns in the PDL rupture behaviour across the full set of extracted teeth.

#### 2.1.2 Statistical analysis of peak forces

Peak force data from successful extractions were normalized by the RSAA obtained with CT imaging and examined for significant differences among groups using a Kruskal–Wallis test in MATLAB with a Tukey’s Honestly Significant Difference *post hoc* test. *Post hoc* effect sizes (Cohen’s D) were estimated for each pairwise comparison of RSAA-normalized peak force as little other biomechanical data is available to contextualize the design of this study. In order to further investigate the relationship between RSAA and peak extraction force, mixed linear models were fit to the peak force, load scheme group, and RSAA data in MATLAB. This modelling method allowed the introduction of random and covariate effects to the ordinal load group data, facilitating a quantitative assessment of the influence of RSAA on peak extraction force while accounting for differences between load schemes. Pairwise comparison of these models with the “lmmodelcompare()” MATLAB command using different combinations of omitted and included terms allowed for the isolation of the most likely effective model. The initial model (most terms) for the analysis is given in Equation 1:

$$Peak\;Force=C+\sum {Β}_{i}X_{Codified\;Group}+\gamma \times RSAA+Zu_{RSAA}+\epsilon$$ (1)

where C is the model intercept; $B_{i}$ the coefficients corresponding to the codified extraction group $X_{Codified\;Group};\;Z$ and $u_{RSAA}$ the coefficients and distribution representing randomness in RSAA; γ the covariant coefficient for RSAA; and ϵ the residual error in the model.

### 2.2 Numerical study

An axisymmetric representation of the dental complex has been previously developed (Gadzella et al., 2024) and was improved upon in current work utilizing expanded data sets towards its implementation in more advanced loading schemes and predictive modeling. The model consisting of the Benex insert, tooth, PDL, and bone (Gadzella et al., 2024) was constructed in the FEBio finite element environment (Maas et al., 2012) to simulate the vertical extraction forces applied in experiments. The material model for the PDL was a visco-damage-hyperelastic model based on the Arruda-Boyce hyperelastic model with strain energy density (SED) function given in Equation 2:

$$\Psi =\mu \sum_{i=1}^{5}\frac{C_{i}}{N^{i-1}}\left(I_{1}^{i}-3^{i}\right)+\frac{1}{2}k{\left(\ln J\right)}^{2}$$ (2)

where $I_{1}$ is the first invariant of Cauchy strain, $C_{i}$ are constants determined from the Taylor expansion of the Langevin expression determined in the model derivation, J is the Jacobian of the Cauchy strain tensor, and parameters μ (initial modulus), N (chain number), and k (bulk modulus) define the material behaviour. The default quasi-linear viscoelastic formulation in FEBio (Maas et al., 2012) was used to represent the viscoelasticity of the PDL based on an exponential relaxation function given in Equation 3:

$$G\left(t\right)=g_{0}+g\;*\exp\;\left(-t/\tau \right)$$ (3)

where $g_{0}=1$ and g is a relaxation constant corresponding to relaxation time t . Finally, the damage of the PDL was governed by a quintic polynomial (Equation 4a) with SED ( ψ ) thresholds $SED_{\max}$ and $SED_{\min}$ determining the limits of the damage function (Equation 4b):

$$D\left(\psi \right)=\left\{\begin{array}{c} 0\;\psi \leq SED_{\min}\\ x^{3}\left(10-15x+6x^{2}\right)\;SED_{\min}<\psi \leq SED_{\max}\\ 1\;SED_{\max}<\psi \end{array}\right.$$ (4a)

$$x=\frac{\psi -SED_{\min}}{SED_{\max}-SED_{\min}}$$ (4b)

when the strain energy density in an element is below $SED_{\min}$ , the stress behaviour of the element is determined only by the elastic (and viscoelastic) response of the base material. Between $SED_{\min}$ and $D_{\max}$ , the stress behaviour is downregulated by the damage variable $D\left(\psi \right)$ , which follows the relationship with $SED_{\min}$ and $SED_{\max}$ given in Equation 4a and ranges from 0 to 1. When the element is completely damaged (at $\left.SED_{\max}\right)$ , $D\left(\psi \right)$ is one and the element can no longer sustain load. This damage model is native to the FEBio software environment.

The result was a set of seven parameters that are used to fit the force-time response of the finite element (FE) model to the experimental data: initial modulus μ , chain number N , and bulk modulus k for the hyperelastic model (Equation 1); relaxation constant g and time constant τ for viscoelasticity; and damage-limiting strain energy density thresholds $SED_{\max}$ and $SED_{\min}$ . Force-time curves for each successful extraction in a single displacement-controlled test group were fit at once, accommodated by the method of aligning each force-time curve at a common transition past 50 N. Outlier curves were isolated and omitted by examining the 95% confidence intervals for the force-time curves of each load group.

The error function minimized in the inverse finite element analysis (IFEA) problem consisted of the product of the sum-of-squares error for a single experimental curve to the finite element analysis (FEA) model response, the square of the peak force difference between the experimental and FEA model curves, and the square of the time-at-peak force difference. These errors were summed for every curve in a load scheme group to achieve a single error function for the group which is minimized using the constrained Nelder-Mead algorithm in MATLAB (D'Errico, 2023) and the GIBBON interface between FEBio and MATLAB (Moerman, 2018). The initial guess for each test group was determined from a range of values available in the PDL modelling literature (Toms et al., 2002; Oskui and Hashemi, 2016; Huang et al., 2016; Genna et al., 2008; Natali et al., 2008; Ortún-Terrazas et al., 2019).

### 2.3 Application of the finite element model to predicting force-hold loading response and the effect of PDL damage

#### 2.3.1 Investigation of force-hold extraction schemes

One of the study objectives was to demonstrate the application of advanced, experimentally-based, loading schemes in developing new extraction processes. Previous work found peak extraction forces at 0.2 mm/min ranging from 102 N to 224 N with no extraction failures, with higher forces and increased extraction failure risk at higher loading rates (Gadzella et al., 2023). It was hypothesized that loading the dental complex to a constant force threshold between 150N and 225 N (near the upper limit of the 0.2 mm/min force range) would result in significant creep displacement due to the viscoelasticity of the PDL, and that the strain energy accrued during creep would be sufficient for successful extraction. To investigate this hypothesis, two force thresholds of 175N and 200 N were selected because they were below the minimum successful extraction force of 244 N identified. Swine incisors were extracted using the established *ex vivo* method to investigate these force-hold schemes. Six incisors for each force threshold (n = 12) were extracted in a randomized order. To limit the test time, force holds were maintained for 500 s before the loading was again increased at 100N/min until extraction. Simulation of this loading scheme using the axisymmetric FEA model predicted that extraction would occur during this constant force-hold period at both 175N and 200 N force-hold levels.

#### 2.3.2 Investigation of damage influence on force-hold extractions

A secondary set of experiments were conducted to further demonstrate the application of both numerical and experimental methods for the predictive modelling of different tooth extraction scenarios. The damage scenario selected for this section of the study is that imposed by the insertion of a flexible periotome (TBS Dental, Union, NJ, United States) inserted into the PDL space to an approximate depth of 4 mm. Insertion of a periotome to both the apical and gingival ends of the PDL space was studied. Gingival insertion of an instrument to the PDL space is a common method for severing the PDL before extraction (Sharma et al., 2015; Contractor et al., 2023) but the biomechanical effect of this action has not been closely examined. Insertion of a periotome to the PDL space from the root apex is not typically possible in clinical cases but was available in this experimental study due to the sectioning of mandibles during sample preparation. Although not clinically representative, this damage case was studied as an analogue for apical decay, resorption, or trauma.

**FIGURE 1:**
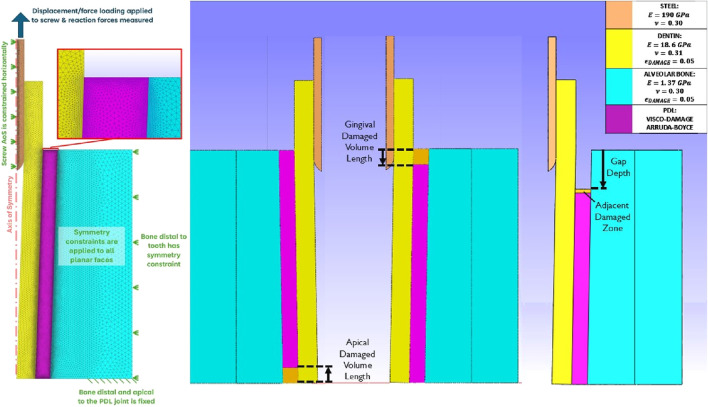
Planar views of the axisymmetric dental complex model. Left: description of the mesh and conditions described in (Gadzella et al., 2024); Centre: damage PDL volumes at the apical and gingival sides; Right: Damage represented as a gap with adjacent zone in the gingival PDL.

Damage occurring from periotome insertion was modelled in FEA simulations as zones of elements with reduced damage capacity relative to the rest of the PDL. The PDL body was remeshed to contain a damaged zone with a depth of 2.5, 5, and 7.5 mm to represent a range of damaged volumes that could result from the periotome insertion, with damaged volume depth defined from the apical or gingival extent of the bone body (Figure 1). Damage was induced in these zones by creating a gradient in the damage-limiting $SED_{\max}$ from the outer edge to the main PDL body using the field generation tool native to FEBio. Modeling damage in this way reduced both the total energy capacity of the elements in the damaged volume and their compliance *via* the shaping of their stress curves with varying limits of the quintic polynomial given in Equation 4b. The value for $SED_{\max}$ at the outer edge was tested at 0%, 50%, or 75% of the nominal value from the IFEA experiments. The model displacement-time response for each of the three damage depths and threshold levels was simulated for both the apical and gingival damage cases (total of 18 simulations) with a 100 N/min ramp to a force threshold of 175 N. Following the initial investigation, additional meshes were generated for modelling damage to the gingival margin of the PDL by creating gaps in the mesh at depths of 2.5, 5, and 7.5 mm. In these meshes, the damaged zone depth was maintained at 0.25 mm. Damage thresholds were manipulated over the same range from 0%–75%.

**FIGURE 2:**
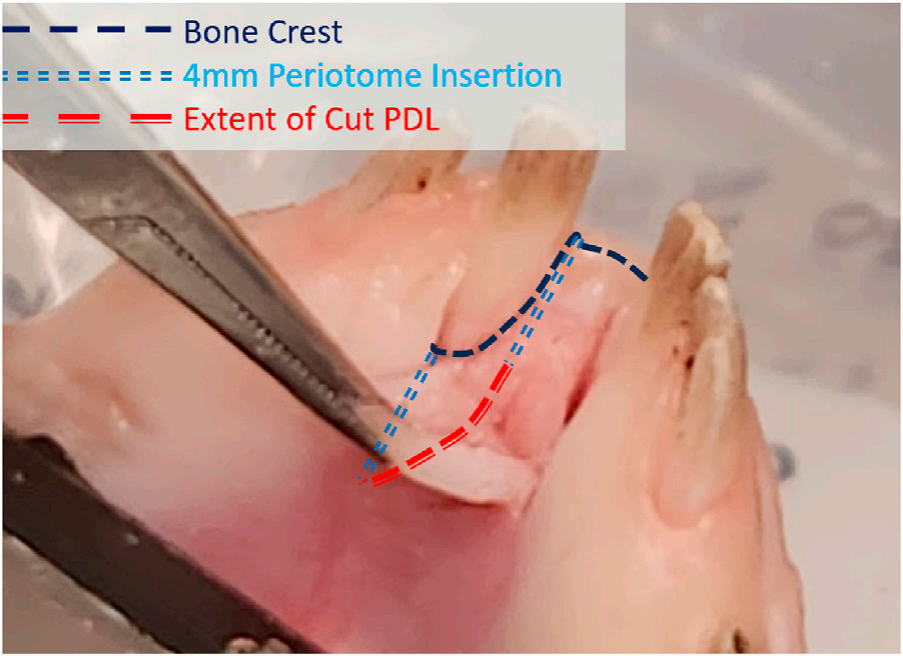
Diagram of the apical margin of the alveolar bone with path followed by the insertion of a flexible periotome to a depth of 4 mm.

Experimental extractions were performed to examine the predictive capability of the FEA model in the damaged cases. The flexible periotome was marked to the desired insertion depth of 4 mm with a permanent marker. In the apical damage case, the periotome was inserted to this depth following the circumference of the exposed periodontal space. In the gingival damage cases, a scalpel was used to create a gingival flap that was peeled back with tissue forceps to expose the alveolar bone crest around the central incisors. The periotome was then inserted to the 4 mm depth following the shape of the alveolar crest around the incisor (Figure 2). Three tests were performed for each of the apical and gingival damage cases (total of 6) at a 175 N target force threshold.

## 3 Results

### 3.1 Mechanical tooth extraction experiments

**FIGURE 3:**
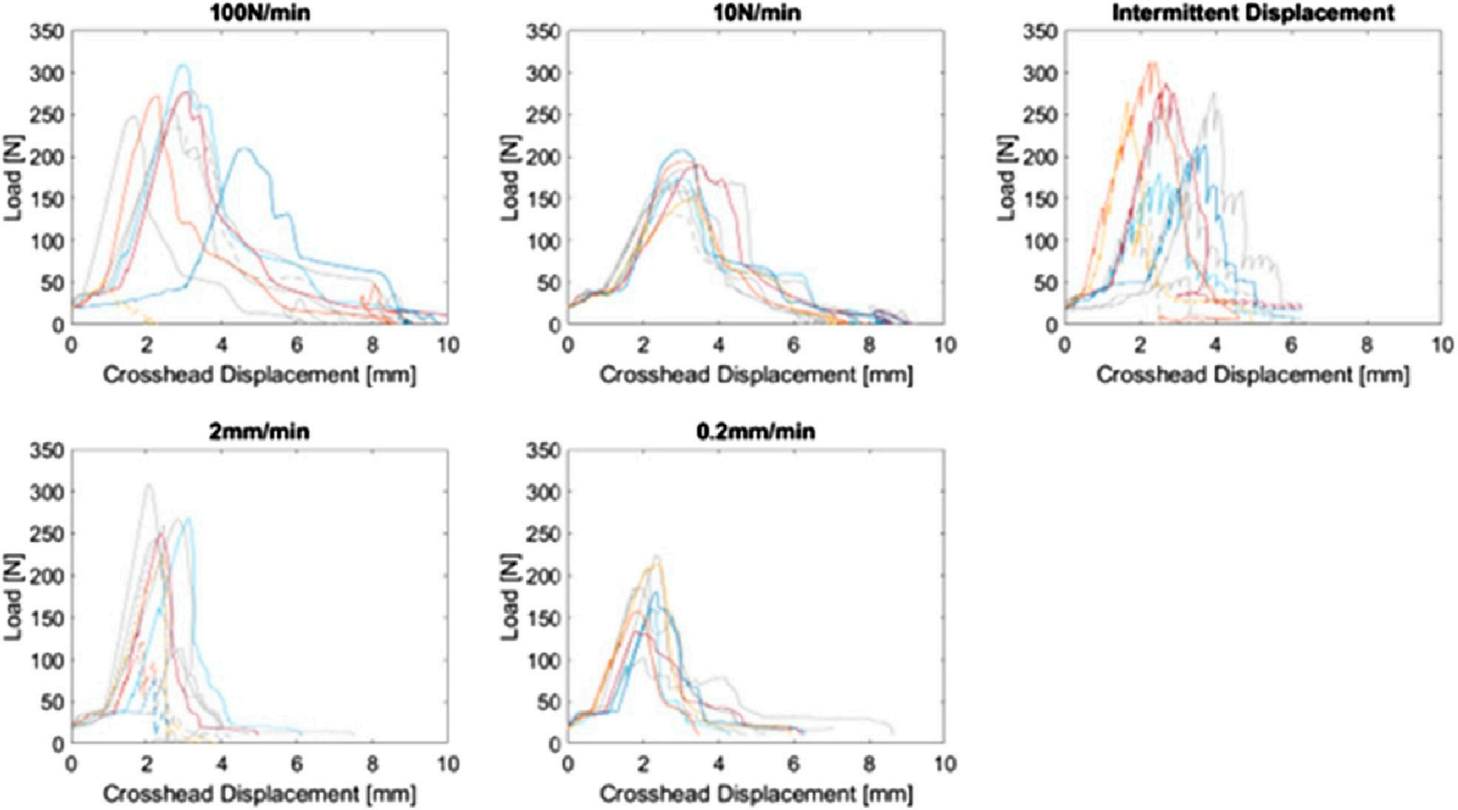
Crosshead force-displacement curves for the complete study (n = 49), plotted by load scheme group. Curves from the previous study (Gadzella et al., 2023) are coloured grey. Dashed curves indicate failed extractions due to tooth or bone fracture.

Upon completion of *ex vivo* experiments, one extraction was omitted due to a failure of the interface between the Benex^®^ extraction screw and the tooth root that was determined to be unrepresentative of a clinical extraction failure (i.e., mechanism failure vs successful extraction or failure of the dental complex). The crosshead force-displacement data from these tests is demonstrated in Figure 3.

#### 3.1.1 Statistical analysis of peak forces

**FIGURE 4:**
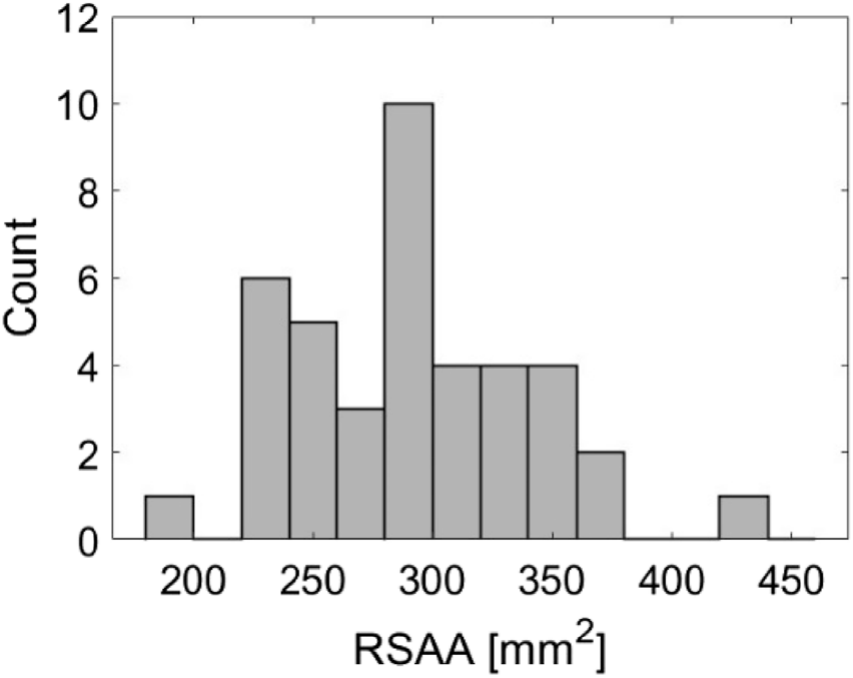
Histogram demonstrating the distribution of root surface attachment areas (RSAA) across 40 successful *ex vivo* extractions.

The distribution of RSAAs for successful extractions with *post hoc* imaging is demonstrated in Figure 4. RSAA values range from 183mm^2^ to 434 mm^2^. The distribution appears to be approximately symmetrical about an RSAA of 300 mm^2^ and does not appear to contain any systematic trends (e.g., skew or multiple peaks/means) that would influence the normalization of peak forces.

**FIGURE 5:**
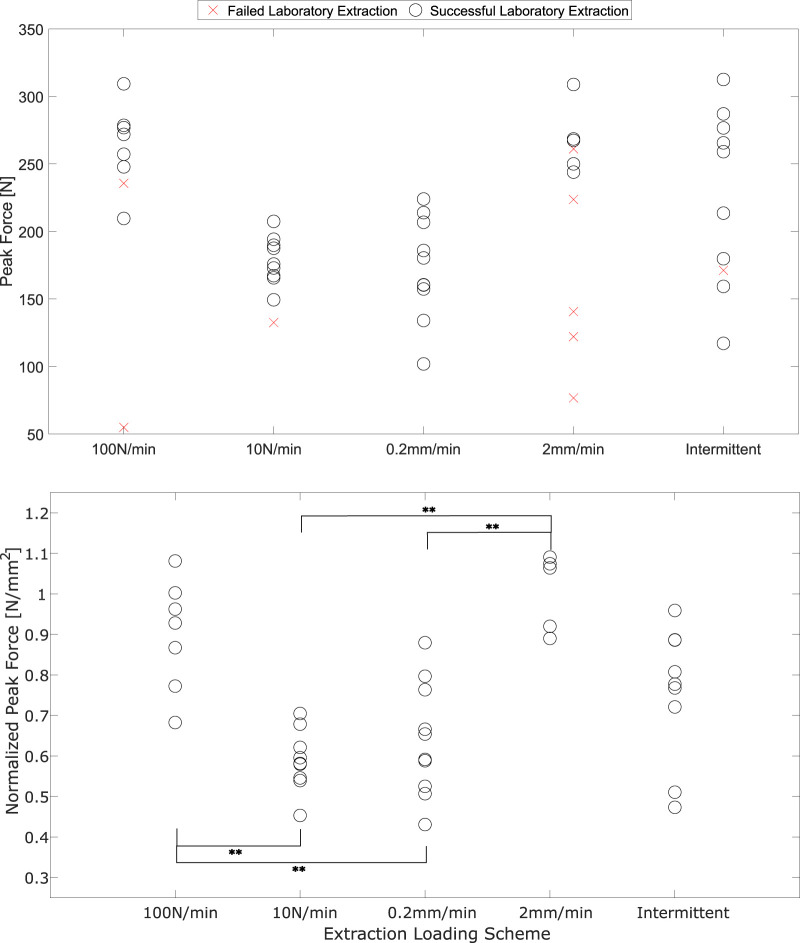
Peak forces from *ex vivo* tooth extractions, un-normalized and including failed extractions (above) and normalized to RSAA for successful extractions. ** - comparisons with significant differences determined by Tukey HSD *post hoc*, *p* < 0.05 Cohen’s D effect size estimates ranged from 0.43–4.46. The lowest effect size estimate (0.43) was calculated for the comparison between 10 N/min and 0.2 mm/min. All other effect size estimates exceeded 0.70.

Peak forces from successful extractions gathered from each test group are compared in Figure 5. An overall significant effect of load group was found among normalized peak forces for successful extractions (*p* < 0.01). The Tukey HSD *post hoc* test found significant differences between both 2 mm/min and 100 N/min load groups when compared individually to the 10N/min and 0.2 mm/min groups (*p* < 0.05). No groups were found to differ significantly from the intermittent displacement loading group because the distribution of these peak forces spans the range of peak forces from all other groups, even when normalized to RSAA. The greatest number of failed extractions was in the 2 mm/min load case (4), with two failures in the 100 N/min case and one each for 10 N/min and the intermittent loading.

The linear model predicting peak force determined by the comparative analysis had a reported *R* ^2^ = 0.623 and is represented by:

$$Peak\;Force=C+\sum {Β}_{i}X_{Codified\;Group}+\gamma \;*\;RSAA+\epsilon$$

where C=140.9N

$$B_{i}=\left[-108.3N,-98.3N,-55.8N\right]$$

$$X=binary\left({\left[\begin{array}{ccc} \frac{10N}{minute} & \frac{0.2\;mm}{minute} & Intermittent \end{array}\right]}^{T}\right)\text{ and }\gamma =0.475N/mm^{2}$$

**FIGURE 6:**
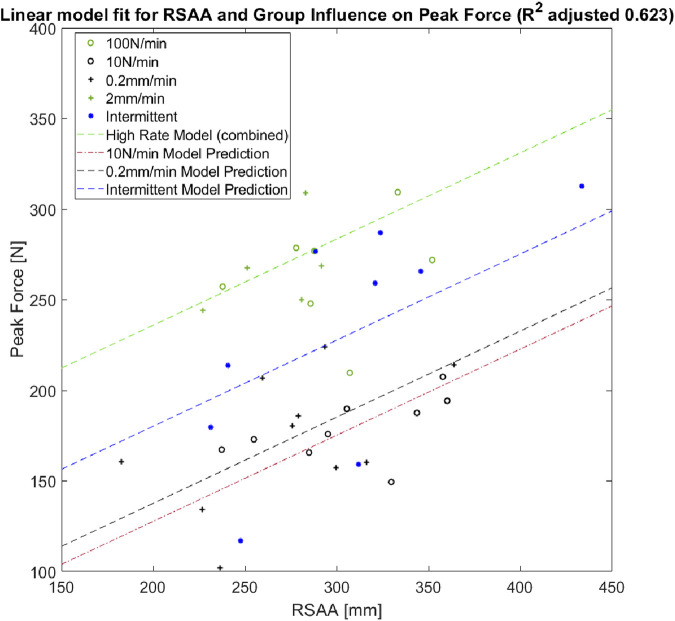
Scatter plot of RSAA and peak force for successful extractions. Linear model is represented for each significant loading group with RSAA covariance.

Similar to the comparison among normalized groups, the linear model analysis also grouped the 2 mm/min and 100 N/min groups together (represented by the intercept constant C ). Each of the 0.2 mm/min, 10 N/min, and intermittent test groups were determined to have the individual coefficients $B_{i}$ . The effect of RSAA variations is represented by the covariate coefficient γ of 0.475 N/mm^2^. The fit of this model to the peak force and RSAA data is presented in Figure 6.

#### 3.1.2 Stiffness analysis

**FIGURE 7:**
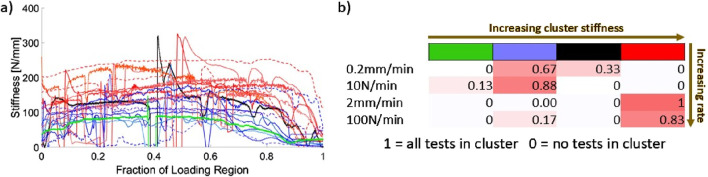
**(A)** Instantaneous stiffness curves, sorted by cluster obtained from the K-means algorithm. Fraction of loading region is from video-identified onset of tissue loading to peak force; **(B)** Likelihood of a stiffness curve being sorted into a given cluster (identified by cluster colour and number).

21 successful continuous-loading extractions with successful video analysis (points tracked throughout loading without marker occlusion) were identified in this study. Figure 7 demonstrates the results of the K-means analysis and the likelihood of an extraction from each load group being sorted into a given cluster.

The K-means analysis clearly identifies two primary clusters of stiffness with two single-curve clusters interspersed. The higher of the two clusters (red in Figure 7) consists entirely of extractions performed at 2 mm/min and 100 N/min. The lower of the main clusters (blue in Figure 7) is comprised mostly of 0.2 mm/min and 10 N/min extractions with a single associated 100 N/min stiffness curve. Both clusters demonstrate a trend in which the stiffness increases over time before dropping towards zero at peak load. This trend is more pronounced for the higher of the two main clusters, with larger decay ranges for the lower cluster. The rise-and-fall pattern is most pronounced for the 100 N/min curve among those in the lower main cluster.

#### 3.1.3 Post-extraction imaging of tooth surfaces and residual PDL

CE-µCT was used to quantitatively assess the patterns of PDL rupture that occurred during tooth extraction. Images were reviewed by the authors, and no automated analysis tools were used to identify tissue features. Two primary patterns were identified: large areas of denudement of the tooth surface from PDL tissue, and the formation of tissue flaps. Generally, denuded tissue areas occurred in the apical third of the tooth root. Flaps formed throughout the tissue body surrounding the tooth root.

**FIGURE 8:**
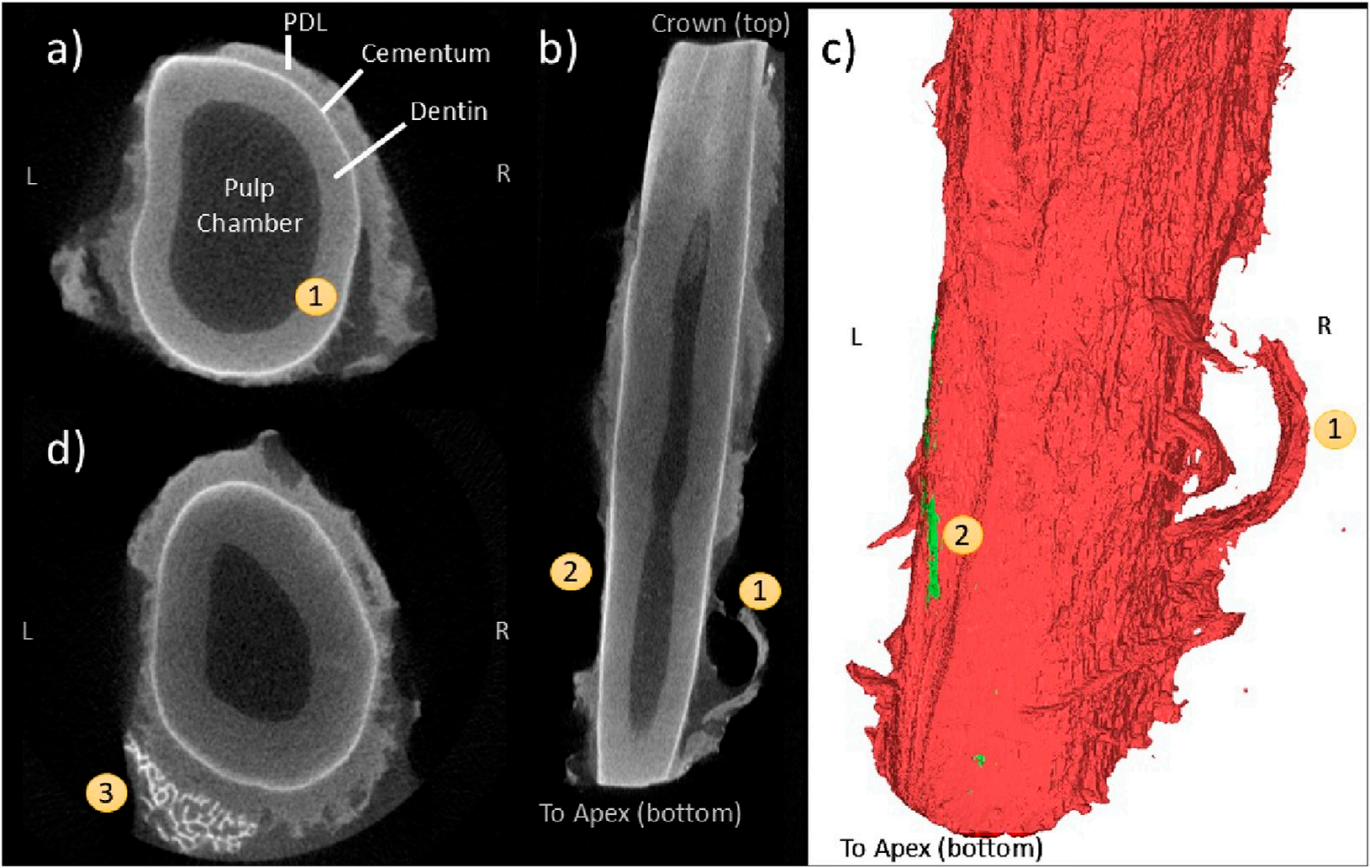
Coronal **(A)**, frontal **(B)**, and 3D reconstruction views **(C)** of a “flap” pattern of PDL rupture (1) observed with CE-µCT. Opposite is an area of root (or tooth?) denudement without a flap (2); A coronal view near the apex **(D)** showing a small fragment of attached bone (3).

The flap pattern appears to originate with rupture at the tooth surface (Figure 8A), extending apically to the root of the flap (Figures 8B, C). There is a significant area of denudement opposite this flap (Figures 8B, C). Immediately apical and adjacent to this area is a small volume of detached alveolar bone (Figure 8D) that was not visible during initial post-extraction assessment. This is the only instance of bone remaining attached to the tooth that was observed in this study.

**FIGURE 9:**
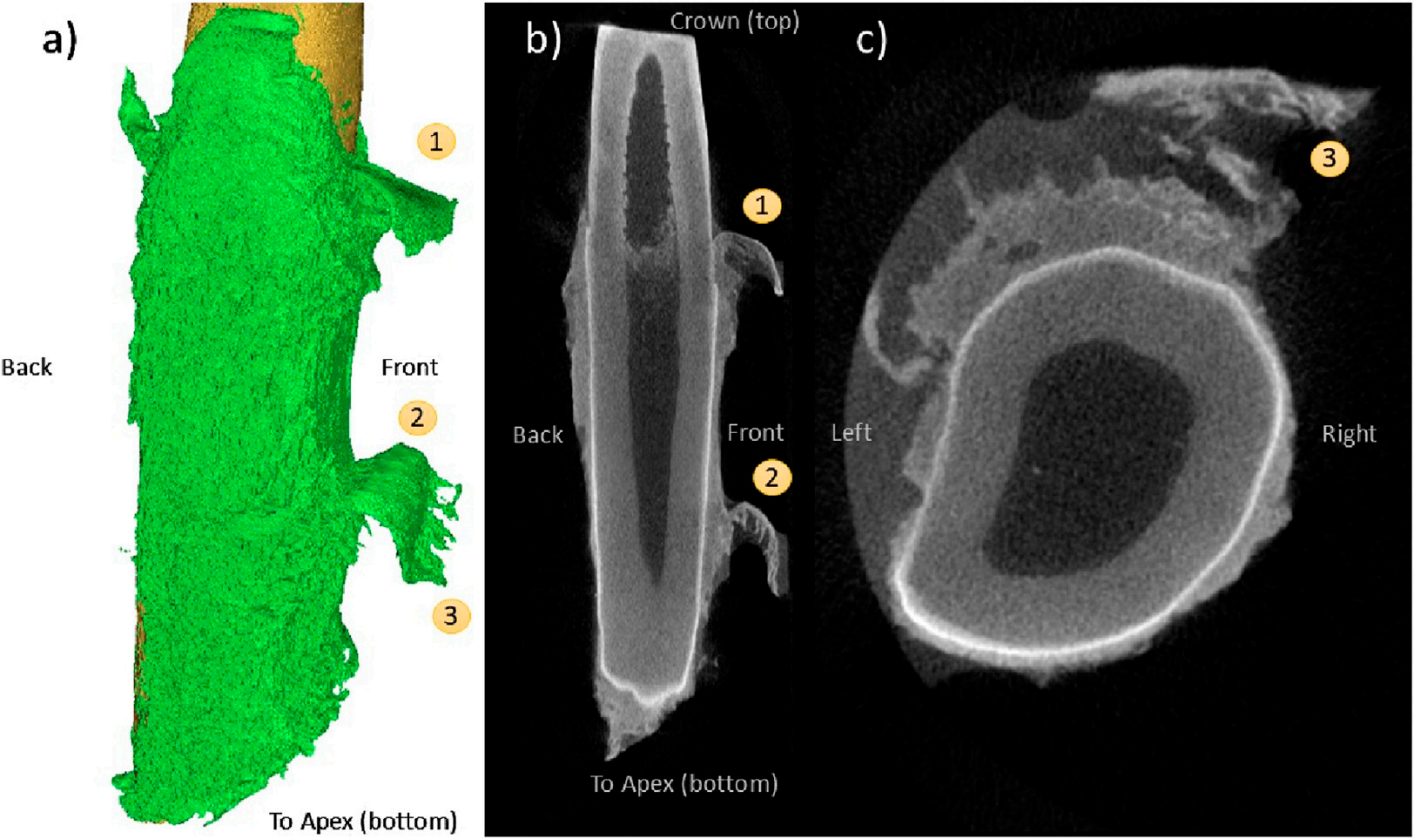
CE-µCT 3D reconstruction view **(A)** and saggittal view **(B)** of two co-linear instances of the “flap” pattern, one gingival occurrence (1) and one mid-apical (2). Fibrous patterns (3) are visible in the coronal view of the tooth **(C)**.

Figure 9A also demonstrates the flap phenomenon, with two pronounced flaps evident on the buccal side of the tooth. The gingival-most flap appears next to the partially denuded cemento-enamel junction (CEJ). The apical-most flap appears adjacent to a region of partial but incomplete tissue thickness reduction (Figure 9B) and demonstrates fibrous strands that appear to be the result of tissue rupture (Figure 9C).

### 3.2 Numerical study

The PDL parameters resulting from the IFEA study are demonstrated in Table 1. There are strong similarities among parameters determined from each of the three load cases. Both viscoelastic parameters and chain link number, N , vary about the coefficient average by less than 10%. The initial modulus values, μ, vary by less than 12% from the average. Bulk modulus k and $SED_{\max}$ vary more (up to 28%), especially in the intermittent loading case. The coefficient averaged solution reflects this similarity across all parameters.

**TABLE 1:** PDL material parameters obtained from IFEA solutions for three displacement-controlled loading schemes.

	g	τ	μ	N	k	$SED_{\min}$	$SED_{\max}$
0.2 mm/min	0.562	21.9	0.422	10.7	7.86	0.696	5.04
2 mm/min	0.520	19.6	0.502	10.2	9.78	0.111	6.22
Intermittent	0.455	19.9	0.470	10.3	11.0	0.195	7.53
Averaged Solution	0.512	20.5	0.464	10.4	9.55	0.334	6.26

**FIGURE 10:**
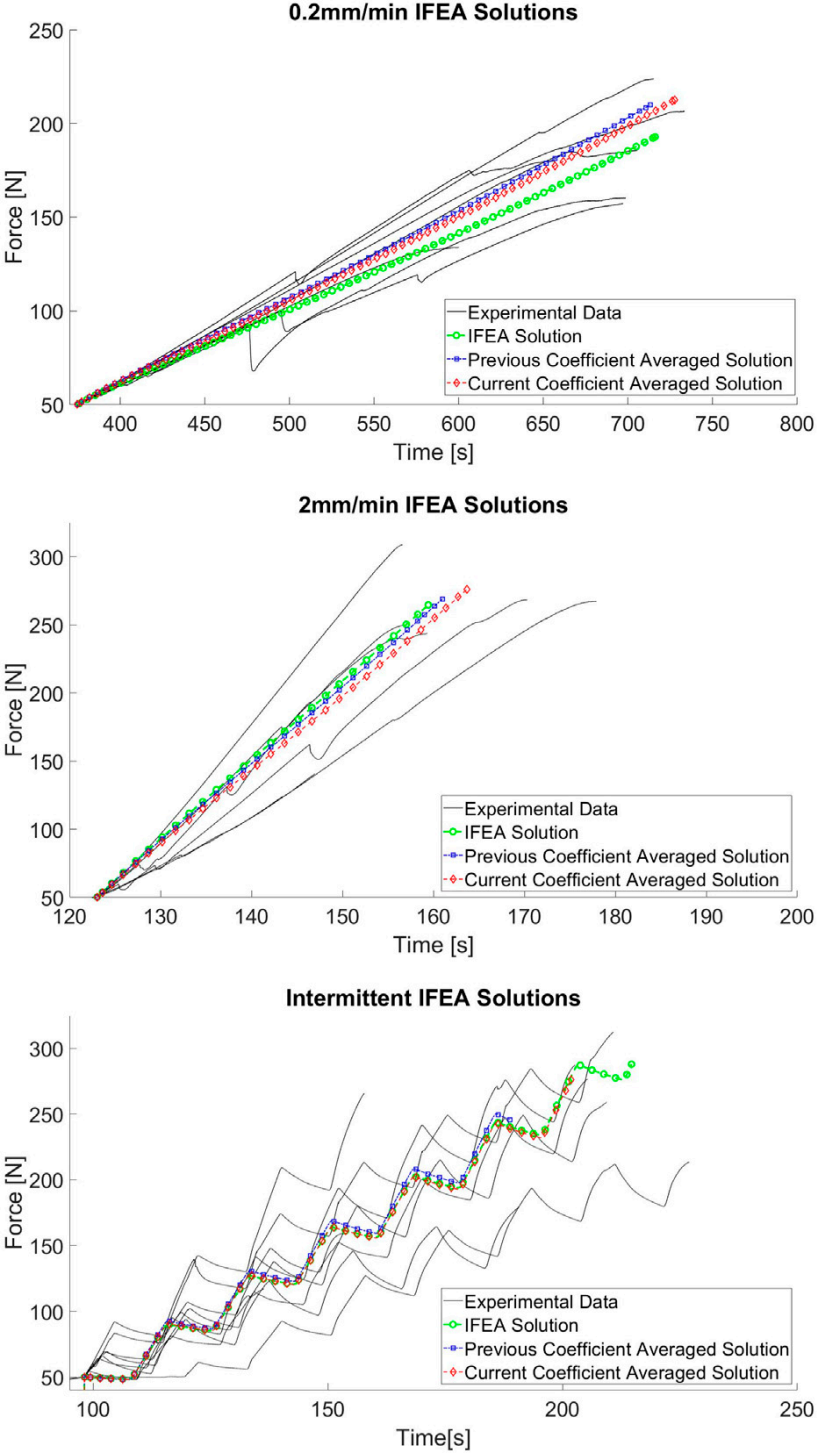
Force-time trace comparison among IFEA solutions and experimental data for 0.2 mm/min **(A)**, 2 mm/min **(B)**, and intermittent displacement **(C)** cases.

The variations in PDL parameters among the IFEA solutions and the current averaged solution are evident in the force-time responses compared in Figure 10. Across all three cases, the performance of the IFEA solutions is good at explaining the overall distributions of the experimental data. For each load case, there is a slight variation in FEA response among the IFEA solution and the two coefficient-averaged solutions in terms of both curve shape and peak force. Differences in curve shape are most pronounced in the 0.2 mm/min group, with the IFEA solution underpredicting both averaged solutions. The differences in time-to-peak-force and peak force are most pronounced in the intermittent loading case, with the coefficient-averaged solutions predicting earlier rupture and lower peak force than the IFEA solution as a result of their relatively reduced $SED_{\max}$ values.

### 3.3 Investigation of force-hold extraction schemes

**FIGURE 11:**
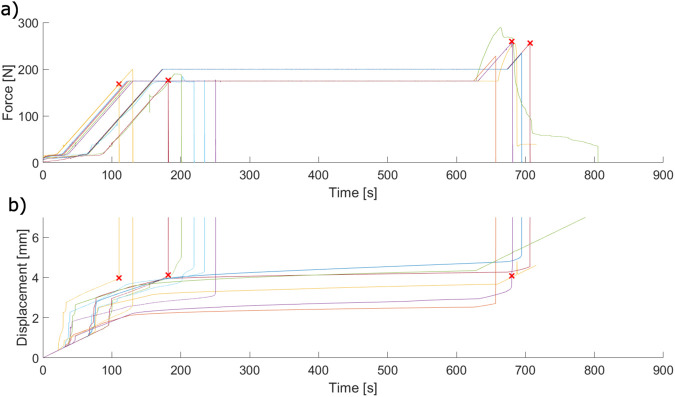
Force-time **(A)** and displacement-time **(B)** crosshead data collected during force-hold *ex vivo* extractions. Each pair of coloured curves is a single extraction. Red “X” marks indicate the point at which an extraction failed due to tooth root fracture.

Crosshead data collected during the 175 and 200 N force-hold tests (Figure 11) demonstrates the self-alignment behaviour of the apparatus at low forces, indicated in the large millimeter-scale displacements that occur concurrently with periods of low force. After system alignment, the dental complex response to the force-hold loading can be categorized into three categories: tests which ended during the initial 100 N/min ramp; tests which ended during the force-controlled hold; and tests which ended during the post-hold increase in loading. Tests ended due to either tooth extraction or fracture of the tooth root in all three categories (indicated by red markers).

The displacement-time behaviour of the force-hold extractions demonstrates creep in varying degrees. Some extractions appear to reach equilibrium within the 500 s hold period, whereas others continuously displace and do not reach this equilibrium state before the end of the period. This behaviour does not appear to depend on the force threshold applied.

### 3.4 Investigation of damage initiation influence on force-hold extractions

**FIGURE 12:**
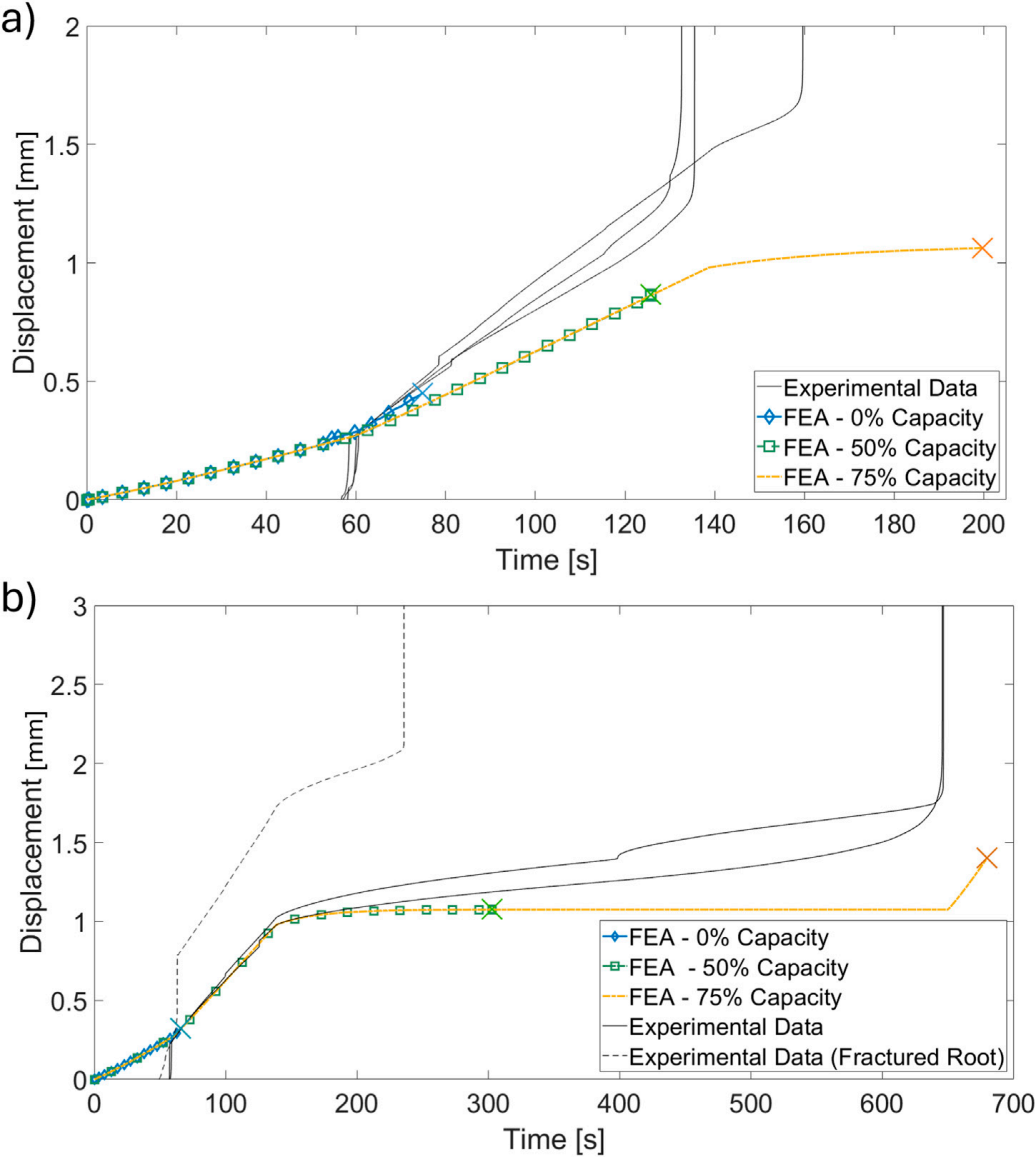
Comparison of displacement-time traces gathered with experimentally-modeled damage of the **(A)** gingival PDL and **(B)** apical PDL compared to finite element simulations. “X” markers indicate PDL rupture ending the simulation in finite element results.

Displacement-time data from both the experiments and FEA models of PDL damage are demonstrated in Figure 12. The FEA study demonstrated that the time of rupture depended strongly on the depth and capacity of the damaged PDL volume, but the shape of the displacement-time curve (i.e., dental complex stiffness for a given constant force-loading rate) did not. Only FEA curves from the 4 mm damage volume depth are demonstrated for clarity–all other curves directly coincided with these curves with varying end points. All experimental curves are adjusted to share a common transition time from apparatus self-alignment to tissue extension with the FEA results.

When the gingival PDL was damaged, only one experiment lasted to the 175 N force hold (Figure 12A). Other extractions were completed during the 100 N/min ramp just prior to the force-hold transition. The FEA results underpredict the experimental displacements but demonstrate a range of rupture times that capture the experimental extractions.

One experimental extraction in the apically-damaged PDL case failed to due fracture of the tooth root (Figure 12B). The other two experimental extractions demonstrated continuous creep over the force-hold period before extraction just prior to the end of the 500 s period. The finite element results predicted extraction in all three phases - initial loading, force-hold, and post-hold loading - at 0%, 50%, and 75% damage capacity respectively. Again, the dependence of the system stiffness on damage capacity for this fixed volume appears to be low and the FEA results underpredict the displacement in the experimental model.

**FIGURE 13:**
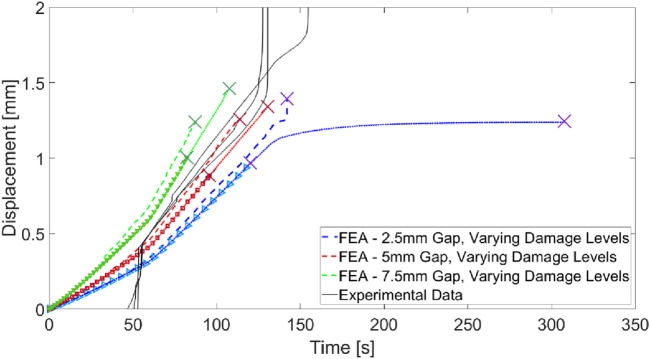
Comparison of experimental force-hold displacement curves for apically damaged PDL with FEA results representing gaps imposed at the damage site. Varying line types within a single colour (red, green, or blue) represent varying SED capacities in the gap-adjacent damaged PDL “X” markers indicate PDL rupture ending the simulation in finite element results.

Introducing a gap at the apical edge to represent damage better simulates the stiffness of the experimental model, evidenced by the alignment of the experimental curves with the FEA results (Figure 13). Introducing the gap also introduces a slight sensitivity in curve shape to the damage capacity but changes in stiffness are dominated by the gap depth.

## 4 Discussion

The purpose of the presented study was to characterize the biomechanical response of the dental complex to vertical tooth extraction and demonstrate the application of a relevant numerical model to predict *ex vivo* model response under previously unexamined loading scenarios. The resulting models demonstrate that peak force and stiffness depend on load rate; that tooth geometry represented by RSAA also determines peak force; and that a single set of PDL material parameters gathered from independent load case IFEA solutions reasonably predict the force-time response of the dental complex to displacement-controlled vertical extraction loading. The application of the FEA model as a predictive tool was demonstrated by predicting the displacement-time behaviour of the *ex vivo* experimental model under force-hold loading. Simple changes to the mesh geometry and material parameters were also demonstrated to predict the effects of imposing mechanical damage to the PDL on dental complex biomechanics during vertical tooth extraction.

The addition of imaging measures for RSAA and increased sample size, expanding on previous work (Gadzella et al., 2023), provided important additional data to the characterization of tooth extraction. Significant differences among the peak forces measured in higher- and lower-rate continuous loading groups were found after normalization by RSAA. A mixed-methods model analysis returned a simple linear model for peak force based on loading rate, including the intermittent loading group, with a covariant for RSAA. This finding is consistent with an intuitive understanding of PDL viscoelasticity in that energy dissipated by intermittent relaxation of the tissue may reduce the overall peak force, but still results in higher peak force than a continuous quasi-static load. The description of peak extraction force portrayed by these models clearly illustrates the importance of load rate in determining peak extraction force, aligning well with the viscoelasticity of the PDL which has been extensively characterised in other loading regimes (Natali et al., 2008; Ortún-Terrazas et al., 2019; Ovy et al., 2022; Su et al., 2013).

The quantification of the influence of load-rate dependence with the inclusion of individual tooth geometry (RSAA) is an important finding of this study. Previous clinical measurements of extraction forces and RSAA have shown a moderate correlation during non-prescribed loading applied with a Benex^®^ device (Dietrich et al., 2020). The linear model proposed in this study quantifies the importance of geometry relative to load rate: the range of RSAA (183mm^2^–434 mm^2^) corresponds to an approximate change in peak force of 120 N based on the given covariant coefficient. Reducing the loading rates by a factor of 10 results in an anticipated 98.3–108 N reductions in peak force, indicating that both RSAA and loading rate are factors of similar importance for predicting peak force. Notably, the range of RSAAs measured in this study is consistent with those measured for single- and multi-rooted teeth in a clinical study utilising the Benex^®^ device (52–400 mm^2^) (Dietrich et al., 2020). Given that RSAA is a relatively straightforward measurement of the tooth surface, comparison of the RSAA ranges between the two is appropriate despite the anticipated differences in tooth orientation and periodontal width between species. The inclusion of multi-rooted teeth in the previous human study (Dietrich et al., 2020) indicates that the role of other geometric parameters may be of further interest in characterising the relationship between tooth geometry and extraction biomechanics towards clinical applications. Examples of such parameters may be the width of the periodontal space, approximate curvature of the tooth root, or the relative height, depth, and width of bifurcations in multi-rooted teeth. The presented study also omitted the role of PDL attachment at the apex by truncating the tooth roots. Future work may expand on the presented experimental and imaging methods to characterize these parameters, and should investigate the sensitivity of the FE model to their inclusion.

The results of this study also indicate good agreement between the peak forces measured in the *ex vivo* swine model (102N–309 N) and the range measured in a human clinical study (41 N–629N, Dietrich et al., 2020). This degree of similarity is promising for the applicability of the *ex vivo* swine model in predicting the biomechanics of human teeth, but further work is required to truly demonstrate similarity between the two. The forces applied during clinical extraction are at the discretion of the operating clinician and are based on a complex set of haptic, auditory, and visual feedback during the procedure as well as pre-operative assessment of radiographs. Direct extrapolation of the results of this study to the clinic should be approached cautiously and only on the basis of the measured trends. For example, the differences among peak forces in this study are measured with sufficient statistical power that a similar comparison can be made confidently in a clinical study, Similarly, the trend for instantaneous stiffness differences among lower and higher rates of loading should be anticipated in the clinical case. However, a direct comparison of quantities and magnitudes (particularly force-hold regime targets) between this study and the human clinical case requires further investigation.

The findings of the instantaneous stiffness analysis presented in Figure 8 further support the characterisation of dental complex biomechanics and their dependence on load rate. The K-means clustering algorithm is blinded to both RSAA and underlying load rate but is able to categorize the curves based on high- or low-rate loading based only on the curve features themselves. Instantaneous stiffness is an important characteristic of dental complex biomechanics because it can be continuously monitored during an extraction with the appropriate instrumentation, whereas peak force as a measure relies on completion of the extraction. This finding has practical implications for future development of vertical tooth extraction load schemes and devices. For example, the results of this analysis may be interpreted to provide a stiffness threshold that is used to limit the loading rate applied with an electromechanical control system. The continuously increasing stiffness behaviour and subsequent reduction near peak force highlights the nonlinearity of the system response and the importance of instantaneous measurement and feedback in the future development of extraction methods. In the context of immediate pre-clinical investigation, these findings also suggest that instantaneous stiffness measurement is an appropriate technique for studying the dental complex rather than the traditional practice of performing linear fits post-experiment and neglecting some aspects of the measured response (e.g., toe-in regions and/or yield).

The findings of the IFEA portion of this study updated the material parameters obtained in previous work (Gadzella et al., 2024) with a larger data set, providing a greater degree of confidence in the physical realism of the resulting model. The PDL parameters across each load case are similar and the resulting force-time curve shapes explain the experimental force-time data well, further indicating the physical realism of the model. Some inter-load case variability in the IFEA solutions is to be anticipated with the multi-curve method due to the inclusion terms for the peak force error and time-to-peak-force errors from each individual curve (in addition to a traditional sum-of-squares error approach). The quasi-linear viscoelastic (QLV) model employed for the PDL does not capture the previously observed nonlinear viscoelastic behaviour of the PDL (Oskui and Hashemi, 2016; Huang et al., 2016) that is demonstrated in this study in the increases in relaxation that occur throughout intermittent loading data sets. The QLV model may also explain the near-equilibrium creep behaviour observed in the prediction of the force-threshold tests which only partially matches the experimental findings. It is well understood that QLV model’s fit to relaxation data (i.e., the intermittent displacement loading case in this study) may not closely predict the creep behaviour of the same tissue when using model constants from one mode to predict the other (Fung, 1993). However, the QLV clearly captures the differences among loading regimes in this study as evidenced by the performance of the coefficient-averaged model across loading cases and, provides a functional representation of the basic viscoelastic behaviour of the PDL while only contributing two parameters to the optimization problem. Future work on this method can investigate other appropriate viscoelasticity models to better capture both the relaxation and creep behaivours of the PDL that are relevant to tooth extraction mechanics.

Qualitative assessment of CE-µCT images of extracted teeth revealed additional insight into the mechanism of PDL rupture that further indicate that the IFEA solution is replicating the physical behaviour of the PDL during tooth extraction. The peeling and denudement patterns highlighted in this study demonstrate the importance of the hard tissue boundaries in the biomechanics of the PDL during extraction load, particularly as a site of rupture initiation. The pattern of denudement adjacent to large residual tissue thickness is consistent with the cross-sectional images of the human tooth following forceps extraction provided by Baschong et al. in their study of cell viability (2018), although the mechanism of loading and purpose of their imaging differs. Due to the irreversible extension and damage of the tissue imaged in this study with no untested reference images, the CE-µCT reconstructions cannot be used to reliably reconstruct rupture formation and propagation. However, the patterns of flap formation and tooth surface denudement found throughout the CE-µCT images and demonstrated in Figures 8, 9 are indicative of material behaviour that is consistent with the FEA representation. The formation of a flap at the gingival edge of the PDL evidenced in Figure 9 is particularly similar to the FEA model behaviour, which undergoes rupture initiation at this location. Similar patterns of tissue rupture are evidenced in Figures 8B, C, and apically in Figures 9A, B that are not reflected in the FEA rupture pattern *per se* but are indicative that the concentration of strain at this boundary (which the model does reflect) is physically realistic.

Results from FE simulations demonstrated the predictive power of the model by representing the displacement behaviour of the dental complex under constant sustained force, both with the PDL intact and with damage imposed by the insertion of a periotome. The FEA model predicted rupture in both cases during the force-hold period, which is the middle ground of the three behaviours observed in experiments. This may be in part as a result of the QLV model behaviour which, in addition to neglecting some non-linearity of the system, may face inherent limitations when modeling creep behavior using constants fit from relaxation data (and *vice versa*) (Fung, 1993). Additionally, the predictions used the same 15 mm root geometry from the model’s inception despite the experimental data demonstrating dependence on RSAA. With no way to determine RSAA or root geometry without pre-extraction imaging, this geometry was accepted but variations in tooth geometry may be influenced when each PDL ruptured. This may also explain the limited influence of damage volume and threshold in the apical and gingival damaged FEA models.

Modelling damage as a void volume in addition to inclusion of a damaged zone improved the FEA prediction of force-hold response in the apically damaged PDL. A range of gap depths and damage thresholds was modeled *in silico* without means to characterize the exact extent and nature of the damage imposed by the periotome despite the fixed depth of insertion (i.e., it is unlikely that tissue immediately adjacent to the periotome is undamaged by its insertion). Despite this limitation, a range of simple changes to the FEA model geometry and parameters were able to predict the range of behaviours in the apically-damaged experimental model in terms of both displacement rate and rupture time. This sensitivity further demonstrates the robustness of the FEA model as a tool that, with further work and validation, can be extended to model a range of tooth geometries and extraction procedures. The FEA model flexibility exhibited in this study is of particular interest for further understanding the use of periotomes as a procedural step itself aimed at reducing bone trauma (Sharma et al., 2015; Contractor et al., 2023) but, similar to vertical tooth extraction, has not been adequately investigated from a biomechanical perspective.

Despite the limitations of the QLV and generalized geometry, the findings of this study suggest that an axisymmetric finite element model validated with a large, variable body of experimental data is a powerful tool for designing new vertical tooth extraction loading procedures and studying extraction biomechanics in different tissue conditions. The predictive capacity critically reduces the impact and costs of developing new vertical tooth extraction loading schemes. For example, the damage modelling method employed in this study surveyed a large range of damage conditions in a run of solver simulations that was completed in a number of days as opposed to utilizing laboratory experiments taking weeks and using extensive resources. Without data characterising the actual nature of periodontal injury caused the insertion of a periotome, this low-cost approach created a cluster of data that accurately captured changes in both complex stiffness and rupture behaviour demonstrated by the experimental model. A numerical-experimental-numerical modelling development loop utilizing this approach provides an evidence-informed approach to developing atraumatic extraction techniques while reducing obstacles such as cost and computational intensity.

The findings of this study are promising for the application of the *ex vivo* experimental and numerical models towards improving vertical tooth extraction procedures. However, caution is warranted in the interpretation of these findings for the human clinical model. Swine periodontal spaces are generally wider than in humans, potentially influencing the structural compliance measured in this study. Additionally, swine incisors are longer than human teeth, curved, angulated buccaly (forwards and outwards) to facilitate foraging behaviour. The sample preparation method established previous work (Gadzella et al., 2024) sought to address these differences by studying only the occlusal-most section of the incisors and, as a result, the peak force and RSAA findings agree well with human clinical measurements (Dietrich et al., 2020). Future work now needs to focus on confirming the transferability of findings in the relatively inexpensive, low-risk *ex vivo* model to the clinic. For example, piloting the force-hold load schemes developed in this study in a human clinical population using an instrumented Benex^®^ extractor offers a low-risk avenue to confirm the predictive capabilities of the developed models. Additional future work investigating the influence of tissue microstructure, tooth shape, and periodontal disease on extraction may also provide insight into the differences between the human and swine models that can help guide the interpretation of *ex vivo* results towards improving human health.

## 5 Conclusion

This study characterized the biomechanical response of the *ex vivo* swine dental complex to vertical extraction loading at five initial loading rates and presented a finite element model based on this characterization. Experimental characterization demonstrated dependency of both peak extraction force and rising-side stiffness on the applied load rate, with peak force also depending strongly on tooth geometry determined by RSAA. The PDL parameters resulting from IFEA analysis of the experimental data were consistent among load rates and a coefficient-averaged solution predicted the force-time curves among all three studied load cases. The FEA model was able to predict a range of extraction times for force-hold loading in intact and damaged PDL cases. The best predictions of the damaged periodontal response were obtained when the damage was modelled as a void with an adjacent damaged PDL volume. The biomechanical data and numerical model obtained by this study provide both an enhanced basic understanding of the biomechanics of vertical tooth extraction and useful tools for the modelling and development of new extraction devices and procedures.

## Data Availability

The raw data supporting the conclusions of this article will be made available by the authors, without undue reservation.
