# Supplementary material for: Loading rate, geometry, and damage state influence vertical extraction biomechanics in an ex vivo swine dental model
Source: Front Bioeng Biotechnol. 2025 Jan 7;12:1491834. doi: 10.3389/fbioe.2024.1491834 (PMC11745890; doi:10.3389/fbioe.2024.1491834)
Supplement: Supplementary file 1 [file DataSheet1.DOCX]

Supplementary Material S1 – Characterisation of the K-Means Algorithm Clustering Used in Instantaneous Stiffness Analysis

The purpose of the additional K-means analysis is to examine the factors that influence the sorting of tooth extraction stiffness curves by the K-means algorithm. In principle, the K-means algorithm sorts curves by closeness to cluster kernels based on Euclidean distance at every point in the curve domain, thereby capturing differences in both the stiffness magnitudes and overall shape of the curves. However, the reliability of this method at detecting systematic differences among the load rate groups (rather than random variations) has not been examined. The method for this examination is to decimate the data set into finer clusters by increasing the number of clusters from 4 to 6 and then to 8, re-performing the analysis with 50,000 random restarts for every case. The resulting clusters are then plotted separately and colour-coded so that each load rate has its own colour. Systematic differences in stiffness will survive this decimation, with the lower- and higher-stiffness clusters being subdivided without curves crossing over. Clusters resulting from grouping of random variations will not survive decimation, with blending of curves from both primary clusters in the decimated groups.


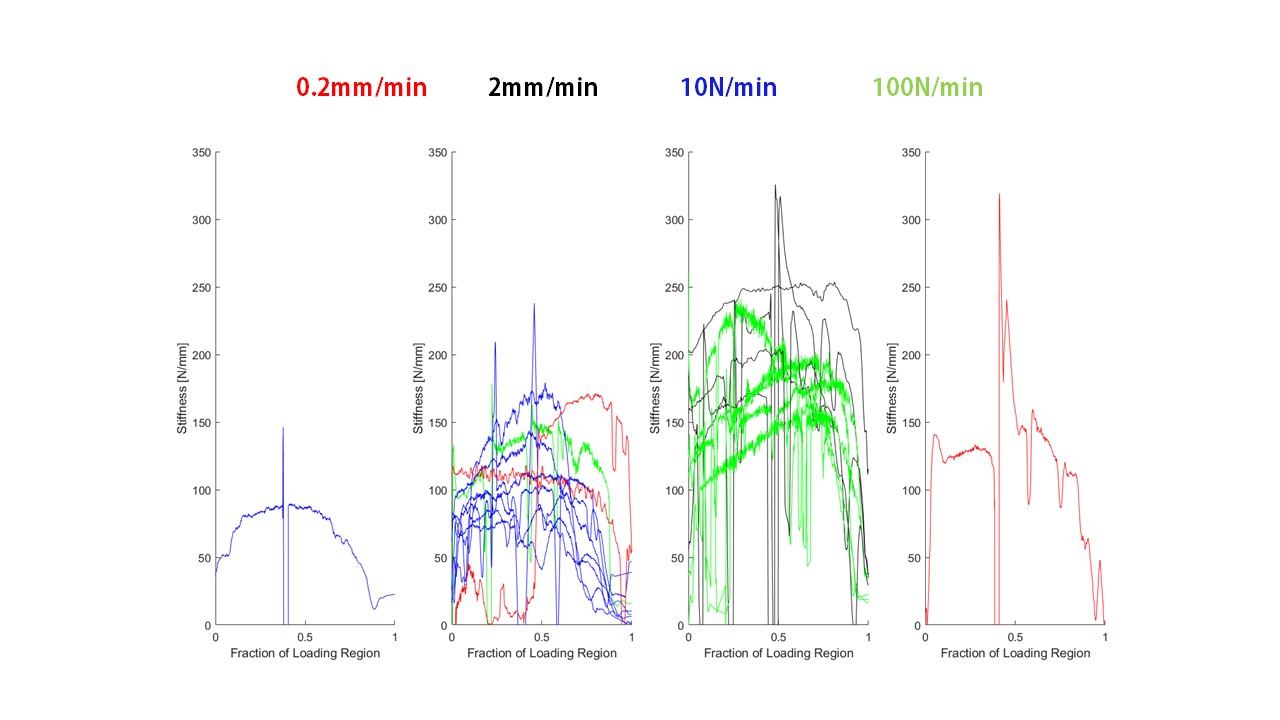


Figure S1: K-Means Stiffness Cluster Results for 4 Clusters. Each sub plot is a cluster with colour identifying the load rate at which a stiffness curve was obtained.

The K-means results for four clusters (Figure S1) demonstrate the sorting of the stiffness curves by load rate. The centre-left curve contains 0.2mm/min (red) and 10N/min curves (blue) with a single 100N/min curve (green) on the higher side of the cluster. The centre-right cluster is the higher main cluster composed entirely of 100N/min and 2mm/min curves (black). Separating the clusters into separate plots also highlights the disruptions that may drive the two single-curve clusters away from the others. Some rapid motion (such as tissue rupture or shift in the self-aligning system) caused large drops and then spikes in stiffness in the two curves that were isolated.


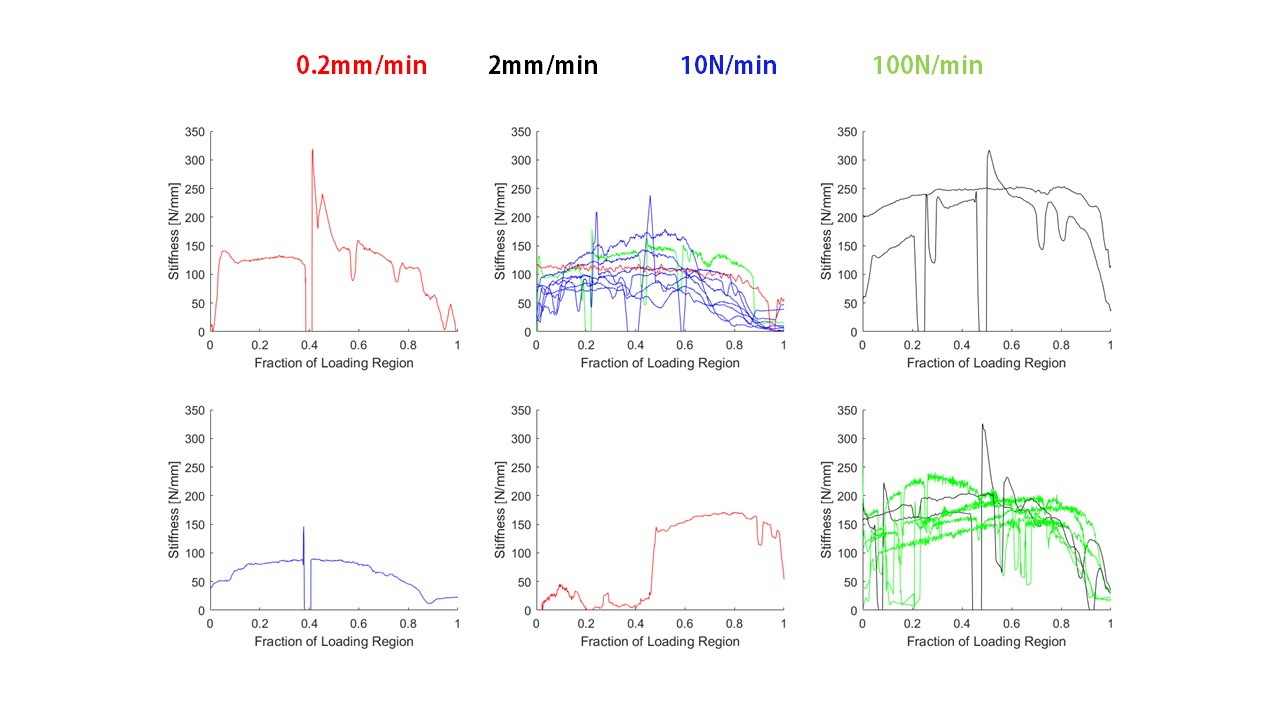


Figure S2: K-Means Stiffness Cluster Results for 6 Clusters. Each sub plot is a cluster with colour identifying the load rate at which a stiffness curve was obtained.

Both primary clusters survive decimation into 6 clusters (Figure S2) by sub-division into two additional clusters. In the higher cluster, two 2mm/min curves are sorted into their own cluster at higher stiffness than the remainder of the cluster. A single 0.2mm/min curve with a prolonged low-stiffness region is isolated from the remainder of the lower main cluster.


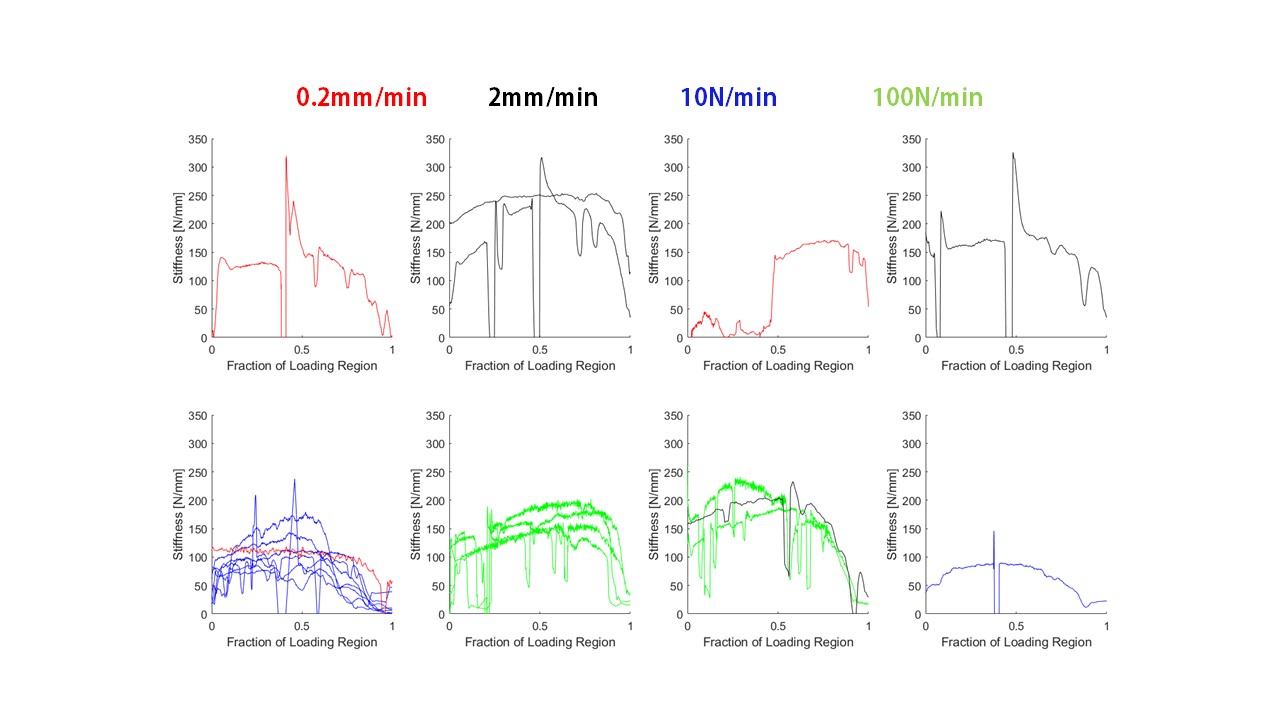


Figure S3: K-Means Stiffness Cluster Results for 8 Clusters. Each sub plot is a cluster with colour identifying the load rate at which a stiffness curve was obtained.

Both primary clusters survived the decimation to 8 clusters with the exception of a single curve, which moved from the lower cluster to the higher cluster. This curve was the 100N/min curve that was sorted into the lower cluster, visible in that cluster in Figures S2 and S1. Additional single-curve clusters are identified along with a split of the higher of the two main clusters.

This analysis confirms that the clusters obtained from the initial 4-cluster K-means algorithm sorting are driven by the systematic differences in stiffness curves based on their underlying load rate rather than random variances. In both decimation steps the clusters are preserved almost perfectly, with only one 100N/min curve moving from the lower main cluster to a higher cluster when the number of clusters in the analysis is doubled. This finding indicates that it is likely random variation in stiffness that caused this curve to be sorted into the lower cluster in the 4-curve analysis but that overall, the differences among load groups are much stronger than these random variances.

Although this decimation analysis shows that systematic differences among stiffness curves can be observed at higher cluster numbers, the results of the 4-cluster analysis are still most appropriate for studying the overall trends in stiffness among load groups. Only four loading regimes are included in the data provided to the K-means algorithm and, although the algorithm is completely blinded to this information, there is no physical basis for the inclusion of more clusters than there are loading regimes. The decimation analysis presented here should be carefully considered as a post-hoc analysis to the four-cluster analysis rather than equivalent in relevance to the physical experiment.
